# Challenges and future directions of AIRR-seq-based diagnostics

**DOI:** 10.1016/j.immuno.2025.100056

**Published:** 2025-07-30

**Authors:** Ulrik Stervbo, Paraskevas Filippidis, Felix Breden, Lindsay G. Cowell, Frederic Davi, Victor Greiff, Anton W. Langerak, Eline T. Luning Prak, Alexandra F. Sharland, Enkelejda Miho, Pieter Meysman

**Affiliations:** a Center for Translational Medicine and Immune Diagnostics Laboratory, Medical Department I, Marien Hospital Herne, University Hospital of the Ruhr-University Bochum, Herne, Germany; b Charité – Universitätsmedizin Berlin, corporate member of Freie Universität Berlin, Humboldt-Universität zu Berlin, and Berlin Institute of Health, Berlin-Brandenburg Center for Regenerative Therapies, Augustenburger Platz 1, 13353 Berlin, Germany; c Department of Pathology, Yale School of Medicine, New Haven, 06511, CT, USA; d Department of Biological Sciences, Simon Fraser University, Burnaby, BC V5A 1S6, Canada; e Department of Health Data Science and Biostatistics, O’Donnell School of Public Health and Department of Immunology, School of Biomedical Sciences, UT Southwestern Medical Center, Dallas, TX, 75390, USA; f Laboratory of Molecular Hematology, Department of Hematology, Hôpital Pitié-Salpêtrière, APHP, Sorbonne Université, Paris, France; g Department of Immunology, University of Oslo and Oslo University Hospital, Oslo, Norway; h Imprint Labs, LLC, NY, NY, USA; i Laboratory Medical Immunology, Department Immunology, Erasmus MC, University Medical Center, Rotterdam, the Netherlands; j Department of Pathology and Laboratory Medicine, Perelman School of Medicine, Philadelphia, PA 19104, USA; k Sydney Medical School, Faculty of Medicine and Health, University of Sydney, NSW 2006, Australia; l Institute of Medical Engineering and Medical Informatics, School of Life Sciences, University of Applied University of Sciences and Arts Northwestern Switzerland, Muttenz, Switzerland; m aiNET GmbH, Basel, Switzerland; n Swiss Bioinformatics Institute, Lausanne, Switzerland; o Antwerp Unit for Data Analysis and Computation in Immunology and Sequencing, University of Antwerp, Antwerp, Belgium

**Keywords:** AIRR-seq, Diagnostics, Standardization, Clinical translation, Machine learning, Interpretability

## Abstract

Adaptive Immune Receptor Repertoire sequencing (AIRR-seq) is a promising diagnostic method across various clinical conditions, yet its widespread implementation faces several challenges. This perspective examines the current landscape of AIRR-seq diagnostics and outlines key obstacles and opportunities for advancement. Critical challenges include the need for standardized quality controls, privacy protection under General Data Protection Regulation (GDPR) and Health Insurance Portability and Accountability Act (HIPAA) frameworks, and the development of clinically compatible bioinformatics pipelines. Machine learning approaches offer potential solutions for interpreting complex repertoire signatures, though these models must balance accuracy with interpretability for clinical adoption. Future applications may include early disease detection, prognosis, and monitoring of treatment and vaccine responses. However, successful clinical integration will require sustained collaboration among funding bodies, regulatory agencies, researchers, diagnosticians, and clinicians to establish clear guidelines and expand existing repositories with well-characterized patient samples. The collaborative efforts of the AIRR Diagnostics Working Group and the AIRR Community’s initiatives are working towards unlocking the potential of AIRR-seq in precision medicine and enhancing diagnostic capabilities.

## Introduction

The adaptive immune system is mediated by T and B cells which recognize and respond to diverse antigens. These cells are defined by their expression of adaptive immune receptors (AIRs) in the form of T cell receptors (TCRs) on T cells and B cell receptors (BCRs) on B cells. The collection of cells, each expressing an AIR, forms the AIR repertoire (AIRR). AIRs are a heterodimeric receptor where each chain contains three complementarity-determining regions (CDR) with greater amino acid and nucleotide variability than the more conserved framework regions of the chains [1]. The BCR consists of two heavy and two light chains, while the TCR comprises either an alpha/beta or a gamma/delta chain pair. The BCR light chain may be of kappa or lambda type. The BCR is a transmembrane protein but can be produced as a secreted form known as an antibody or immunoglobulin (IG) [2]. The TCR is exclusively membrane-bound. A defining feature of AIRs is the somatic recombination of different genes in the TR or IG loci during lymphopoiesis occurring in primary lymphoid organs. Through random insertion and deletion of nucleotides at the junctions, the non-templated, hyper-variable CDR3 is created [3]. This region is largely what endows the AIR with its antigen specificity and, together with the V and J genes, creates a unique identifier, which enables monitoring and tracking of clonally related cells [4].

Immune cells respond to antigens, which may be derived from pathogens such as viruses or bacteria, environmental molecules, as in the case of allergies, or variations of self, as seen in autoimmunity, cancer, and transplant rejection. Only lymphocytes bearing an AIR specific for the antigen become activated, expand and differentiate, which alters the size, shape, function and composition of the immune cell AIR repertoire [5]. Using AIR Repertoire sequencing (AIRR-seq) approaches [6], it has been shown that the repertoire dynamics and/or AIR specificity enable discrimination or classification of different diseases, each characterized by unique AIRR-signatures [7,8].

## The AIRR-community and diagnostics WG

The Adaptive Immune Receptor Repertoire (AIRR) Community, part of The Antibody Society, organizes stakeholders from academia, industry, and other sectors for the study, sharing and analysis of TCR and BCR repertoires. The community has already addressed some of the key challenges presented in this perspective including the development of metadata standards for AIRR-seq datasets [9]. Building on this, the MiAIRR Standard was created [10], adding standardized descriptors to represent annotated repertoire sequencing data. In parallel, data repositories using these standards were created, such as the AIRR Data Commons, a distributed system of repositories for public sharing of AIRR-seq data [11]. The AIRR Data Commons can be queried via an application programming interface (API) [12] or using either of two graphical interfaces of the iReceptor Gateway [13] or VDJServer Community Data Portal [14]. The AIRR Community has also defined standards for AIRR software tools and resources to enable rigorous and reproducible immune repertoire research on a massive scale [15]. Through these initiatives, the AIRR Community has been instrumental in making AIRR-seq data FAIR (Findable, Accessible, Interoperable, and Reusable). The adoption of these standards by some industry stakeholders highlights their significance, although broader industry engagement remains a challenge.

Beyond standardization, the AIRR Community is committed to fostering the development of resources and tools essential for AIRR-seq to gain traction in clinical diagnostics. The Germline Database Working Group, for instance, has made significant strides in creating complete and accurate reference sets of IG and TR germline genes [16], which are crucial for the accurate analysis of AIRR-seq data and for ensuring reproducibility and reliability in clinical settings.

The Diagnostics Working Group of the AIRR-Community envisions a future where AIRR-seq plays a central role in clinical diagnostics, particularly in supporting difficult-to-diagnose cases and enabling personalized medicine [17]. By continuing to develop and promote standards, resources, and collaborative initiatives, the AIRR Community aims to bridge the gap between technological advances and their practical application in routine clinical diagnostics.

## Current applications of AIRR-seq in diagnostics

AIRR-seq holds the potential to significantly enhance diagnostic accuracy across a broad spectrum of clinical conditions. These include primary and immunodeficiency-associated lymphoproliferative disorders, lymphoid malignancies, acute and chronic infectious diseases, vaccine efficacy assessment, and autoimmune disorders [6,18]. While there have been large advances in applying AIRR-seq in the area of therapeutic antibodies [19], CAR T-cells [20], and even TCR-T cell therapies [21], diagnostics based on TCR/BCR repertoire features have been lagging behind, albeit not for lack of effort.

The European study group BIOMED-2, which later evolved into the EuroClonality consortium, has been active in AIRR-based diagnostics for lymphoproliferative disorders since the nineties [22]. BIOMED-2/EuroClonality developed and validated a set of primers to evaluate rearrangements of BCR and TCR genes initially by PCR and electrophoresis-based size fragment analysis [23], and more recently using next-generation sequencing [24]. This EuroClonality-amplicon-based protocol can identify malignant clones within a polyclonal sample with a limit of detection of 2.5 %; however, this sensitivity can be drastically improved for tracking minimal residual disease (MRD) when using optimized protocols and adequate sampling [25]. Currently, the only FDA-approved platform for use in different leukemias was developed by the company Adaptive Biotechnologies [26]. For a recent discussion on the use of AIRR-seq in lymphomas, please refer to the work by Fend and colleagues [27].

AIRR-seq is already well-established in the clinic for hematologic malignancies. Turn-around-times for bulk gDNA-based sequencing of IGH with dominant clone calling and/or IGHV gene somatic hypermutation are in the range of 8–12 days from receipt of sample in a high-throughput setting. Turn-around-times can be longer with send-out testing or testing in smaller labs that may require longer times to accumulate enough samples for a single sequencing run. Cost-effectiveness depends on how overall costs are calculated. While the test itself may incur high costs - due to reagent kits, sequencing costs, regulatory requirements, and the need for highly trained laboratory personnel - the performance of these assays can enable faster initiation of treatment (e.g., diagnostics) or earlier treatment modifications, particularly when using more sensitive MRD assays. These changes in clinical management may affect overall healthcare costs and disease burden. However, this remains an evolving area as clinical use cases for immune repertoire profiling assays continue to expand.

In an early proof-of-concept study utilizing patterns in AIRR-seq data to identify exposure to chronic infectious pathogens, bulk TCR repertoire data were used to distinguish Cytomegalovirus seropositive individuals from seronegative individuals [28]. Since then, several studies have investigated the use of AIRR-seq in detecting different conditions, such as infectious diseases [29–35], autoimmunity [36–42], cancers [43–47], and transplant rejection [4,48–52,30,33,34,41,46,53,54]. In addition, several studies have demonstrated the potential of AIRR-based biomarkers to stratify response subgroups and disease outcomes [55–59]. An overview of current developments in machine learning for AIRR-seq-based diagnostics is discussed in a dedicated paper in this issue.

## Regulatory considerations

Regulatory challenges in the clinical application of AIRR-seq extend beyond laboratory protocols to impact health institutions, software (Medical Device Software, Software as a Medical Device), and computational workflows. For Laboratory Developed Tests (LDT) or in-house tests to be used in a clinical setting, the laboratory must be accredited under the relevant regulatory frameworks. In the United States, laboratories must comply with the Clinical Laboratory Improvement Amendments (CLIA) Standard and FDA LDT rule. In the European Union, regulations mandate clinical tests that comply with In Vitro Diagnostic (IVD) requirements, either as CE-IVD marked tests or as in-house IVDs, developed within accredited institutions and laboratories. Clinical-grade assays must meet standards such as the International Organization for Standardization (ISO) 15,189 standard and the Medical Device Coordination Group guidance MDCG 2023–1.

Beyond a foundational quality management system, several critical factors must be addressed in clinical testing. Internal quality controls play a key role in verifying newly acquired AIRR-seq reagents, assessing sequencing run performance, and validating updates to AIRR-seq software pipelines. Different amplification technologies have different levels of sensitivity and accuracy and there is a clear need for standards and controls [60,61]. While benchmark reference standards remain limited, spiked cell line DNA mixtures, plasmid pools, and synthetic sequence pools have been described as useful tools for both laboratory and computational validation [62–64]. The FDA-led BCR sequencing quality control (BCR-SEQC) consortium, with participation from the AIRR Community, was established to address the lack of standardized assay controls and validation methods. The consortium is working on protocols for developing reference materials and datasets for evaluating AIRR-seq-based BCR repertoire reconstruction and is also developing materials for performing benchmarking studies [65]. External quality assessment programs are essential for ensuring that clinical test results are both reproducible and consistent with consensus interpretations across laboratories. Currently, EuroClonality, EuroMRD, the College of American Pathologists (CAP), and UK-NEQAS offer such programs. Standardized reporting formats further support these efforts by defining structured criteria for documenting results and clinical conclusions.

Lessons from other disciplines highlight how standardization has enabled successful translation to clinical care. In clinical microbiology, the implementation of standardized antimicrobial susceptibility testing protocols, such as those from CLSI and EUCAST, has transformed empirical antibiotic decision-making into a data-driven clinical practice [66]. Similarly, in clinical hematology, standardization of flow cytometric measurable residual disease (MRD) assessment in acute lymphoblastic leukemia and other hematologic malignancies through the EuroFlow Consortium has enabled widespread adoption of MRD as a prognostic and treatment-guiding tool [67,68]. In the domain of bulk transcriptomics, the MAQC (MicroArray Quality Control) consortium established reproducibility benchmarks and reference datasets that laid the groundwork for the use of gene expression profiling in FDA-cleared diagnostic assays, such as MammaPrint and Oncotype DX [69]. These examples demonstrate that rigorous standardization, from reagents and protocols to data interpretation, can drive clinical implementation, improve patient outcomes, and foster regulatory confidence. Analogous frameworks are essential for AIRR-seq assays to realize their full diagnostic and prognostic potential in immunogenomics.

A major challenge in the implementation of clinical laboratory testing is stringent data privacy, security, and reporting requirements. While AIRR-seq assays that employ targeted approaches currently avoid some of the privacy concerns associated with whole-genome or whole-exome sequencing, these assays may become more similar to other genetic tests as knowledge and databases expand [70]. For example, correlations between the expression of TCR Vb and Va sequences and HLA alleles could allow for inference of ethnic background [71,72]. In addition, repertoire features may be linked to specific antigen exposures (such as viral infections), diseases or disease outcomes, enabling disease-relevant information and health status to be gleaned from immune repertoire data. Another challenge is the AIRR-seq data itself, as IG and TR germline sequences and haplotypes can be inferred from AIRR-seq data [73–78]. In addition, there is evidence that the loci and constant regions have sufficient interpersonal variation to serve as genomic fingerprints making it probable that AIRR-seq data itself can potentially re-identify individuals [79–81]. Efficient de-identification of sensitive genomic data can be achieved by adhering to data privacy policies commonly applied in clinical settings.

Many bioinformatics pipelines developed for AIRR-seq research fail to meet the stringent requirements of clinical environments. In the United States, digital health and genetic data are regulated by the Health Insurance Portability and Accountability Act (HIPAA), the Genetic Information Nondiscrimination Act (GINA), the California Consumer Privacy Act (CCPA) and the federal Health Information Technology for Economic and Clinical Health (HITECH) Act. While some of these laws provide a regulatory framework for digitization, meaningful use, privacy and patient access to health records, significant challenges remain with respect to interoperability and data security [82].

The GDPR of the European Union defines genetic data as acquired genetic characteristics of an individual that provide unique information about their health, including AIRR-seq data [83]. The GDPR framework requires that any organization processing data from the European Union must comply with its regulations, regardless of who initially made the data publicly available. Furthermore, if an organization within the European Union processes data created outside the EU for purposes different from those for which it was originally collected, it must adhere to GDPR as the entity determining how the data is used. Providers of AIRR-seq assays for clinical use must comply with GDPR, obtain relevant certifications such as ISO 27,001, and adhere to country-specific regulations. Additionally, they must employ dedicated personnel for platform administration, maintenance, and user support to ensure regulatory compliance and data security.

Diagnostic machine learning models are generally categorized as ‘Software as a medical device’ (SaMD) by the FDA and ‘medical device software’ (MDS) by the European Union [84,85]. Both organizations have adopted a risk-based approach to the evaluation of these models. This approach takes into account the disease in question, the weight of the device’s information for clinical decision-making, and the potential risks of inaccurate results. For instance, FDA has three categories each requiring different levels of regulatory control and rigor of evaluations [86]. Models developed and used in a single clinical laboratory can be considered an LDT by the FDA and cannot be distributed to other laboratories [87]. In fact, models distributed to multiple laboratories are not considered LDTs and must be validated for their intended use by the clinical laboratory. Based on this, the most practical approach to integrate AIRR-seq into clinical decision-making is the use of interpretable models that allow expert clinicians to incorporate immune repertoire insights alongside other clinical parameters, ultimately facilitating personalized patient care.

Multiple complementary strategies support privacy-preserving health data analytics without compromising utility. Homomorphic encryption enables computations on encrypted data [88], and multi-key schemes allow secure multi-source analysis [89]. Secure multiparty computation permits joint analysis while keeping inputs private [90]. Differential privacy adds noise to protect against re-identification [91]. Federated Learning trains models locally, sharing only model updates [92], while secure processing environments ensure data remains in controlled, compliant settings [93]. These tools are vital for digital twins—virtual models using multi-omics data to simulate disease and treatment outcomes [94]. Together, they offer a robust framework for advancing diagnostics while safeguarding privacy.

## Future perspectives

AIRR-seq holds tremendous promise in mapping the landscape of past, current, and future immune responses. Through the analysis of repertoire signatures, it may be possible to assess the efficacy and durability of vaccine responses, uncover latent infections, and identify post-acute infection syndromes, distinguishing them from non-infectious conditions. Additionally, it may enable immune monitoring and infection risk stratification in transplant patients and other immunocompromised individuals (e.g., by detecting viral reactivation). AIRR-seq offers transformative potential for advancing broader areas of diagnostics, such as distinguishing between infectious diseases, autoimmune disease flares, and paraneoplastic syndromes. In most fields, AIRR-seq may also provide valuable biomarkers for disease activity and progression and guide personalized therapeutic strategies, particularly in diseases with highly heterogeneous phenotypes, such as systemic lupus erythematosus and lymphomas. It could also serve as an early warning system for epidemics or pandemics by detecting anomalies in immune repertoires.

Disease classification and identification of various signatures require the application of various forms of machine learning. A critical next step for advancing AIRR-seq diagnostics is, therefore, expanding existing repositories with well-characterized patient samples. The establishment of standardized protocols for sample collection, processing, and data sharing, will lead to a more comprehensive knowledge base that supports both diagnostic development and basic research. Such collaborative efforts would be particularly valuable for rare diseases and complex immune disorders where individual institutions may struggle to accumulate sufficient sample sizes for meaningful analysis. These AIRR data repositories should make linking to comprehensive patient-specific clinical metadata and treatment outcomes possible and include longitudinal sampling where possible. Systematic screening efforts should focus on identifying disease-specific signatures in immune repertoires, particularly through the analysis of antigenic specificities and HLA associations, which may require deep sampling of selected individuals and antigen-enriched immune cell populations.This hybrid approach of sampling specific diseases and specific antigen-binding immune cells will provide a basis for more robust diagnostic and predictive models by improving our understanding of immune responses across different pathological conditions.

The implementation of AIRR-seq diagnostics In low or middle income countries, structural challenges such as the need for robust bioinformatics infrastructure, and logistical issues like reliable electricity and internet connectivity [95,96]. Additionally, there is a critical dependence on accurate HLA typing and comprehensive germline gene databases, which are currently underrepresented for non-European populations [16,74,97,98]. This can lead to systematic misassignments and inaccuracies in somatic hypermutation analysis. Addressing these issues will not only enhance diagnostic precision but also advance scientific understanding.

Current processing times generally limit the evaluation of acute diseases but are excellent for early detection, stratification, and monitoring. This capability makes single, centralized laboratories a cost-effective alternative for primary analysis of data (aimed to provide care for the individual) compared to decentralized, hospital-associated diagnostic labs. These centralized laboratories could provide scientists with a wealth of interoperable sequence information through compliance with data reuse standards and be accessible under proper data governance models. A significant challenge with this structure for secondary uses of data could be the dissociation between genomic data and patient metadata, which complicates improvements to diagnostic methods. Linking de-identified medical records with the AIRR-seq data could bridge this gap while adhering to data protection laws and regulations. Despite the advantages of centralized AIRR-seq data repositories, privacy concerns remain significant. Risks include potential re-identification via HIPAA identifiers, secondary genomic analysis, and unique immune repertoires in rare diseases. Solutions such as privacy-preserving record linkage (e.g., EUPID [99]) may help, although they do not fully eliminate risks.

## Conclusion

AIRR-seq offers diagnostic potential extending beyond lymphoid malignancies and MRD. Collaborative efforts from funding bodies, regulatory agencies, researchers, and clinicians are essential to develop and validate new AIRR-seq-based diagnostic tests. On the one hand, there is a need for clear guidelines and standardized quality measures, while on the other hand researchers should ensure interpretability of the proof-of-concept models for a smooth transition to clinical use. In addition, the scientific community should collaborate to expand existing AIRR-seq data repositories with more well-defined patient samples and initiate systematic screening of AIR sequences, epitopes, and HLAs where appropriate. These steps are essential to accelerate AIRR-seq’s integration into clinical diagnostic practice.

## Data Availability

No data was used for the research described in the article.
